# Do non-bone metabolism laboratory indicators differ between men and women with osteoporosis?: A retrospective study based on physical examination data

**DOI:** 10.1097/MD.0000000000044113

**Published:** 2025-08-22

**Authors:** Xiaoqian Kong, Bohan Li, Yan Shi, Zhe Fang, Yixin Li

**Affiliations:** a Health Management Center, The Second Hospital of Shandong University, Jinan, Shandong, P.R. China.

**Keywords:** differences, gender, influencing factors, non-bone metabolic laboratory indicators, osteopenia, osteoporosis

## Abstract

With the global population aging, osteoporosis has emerged as a significant health concern worldwide. Osteoporotic fractures predominantly occur in the forearm, hip, and lumbar spine, and are associated with substantial morbidity and mortality. Osteoporosis is a multifactorial disease, and prior research has identified several clinical risk factors, including advanced age, gender, weight, history of fractures, smoking, alcohol abuse, and corticosteroid use. To ascertain whether non-bone metabolic laboratory indicators associated with osteoporosis vary by gender, this study aims to enhance individual risk assessment, and inform targeted early prevention and treatment strategies for individuals at high risk of developing osteoporosis. Data were collected from a cohort of 4343 individuals who underwent routine physical examinations, bone mineral density (BMD) assessments, and associated laboratory tests at the Department of Health Management of the author’s institution, spanning from January 2022 to June 2024. The study focused on analyzing non-bone metabolic laboratory indicators related to osteoporosis. Unconditional logistic regression was employed to evaluate the risk factors for osteoporosis and osteoporosis in both males and females. The influencing factors of osteoporosis in males are blood urea nitrogen (BUN), and in females, alanine aminotransferase (ALT) and uric acid (UA). The influencing factors of osteopenia in males are BUN, while in females, they are hypertension UA, total cholesterol (TC). Age, weight, and alkaline phosphatase (ALP) are common influencing factors for osteoporosis and osteopenia in different genders, but the importance of weight and ALP varies. The importance of weight was almost equal to age in male with osteopenia, but was significantly less than that of age in female. The importance ranking of influencing factors in male osteoporosis is weight and ALP, while for females it is ALP and weight. The influencing factors of osteopenia and osteoporosis vary between different genders. Even the same factor (weight, ALP) has different degrees of effect on osteopenia and osteoporosis in different genders.

## 1. Introduction

Osteoporosis is a degenerative condition affecting the skeletal system, recognized as a significant global health issue due to the aging population.^[1]^ The disease is characterized by a reduction in bone mass, decreased bone mineral density (BMD), and deterioration of the bone microarchitecture, leading to increased bone fragility and a heightened risk of fractures.^[2]^ According to a meta-analysis study, the global prevalence of osteoporosis and osteopenia is 19.7% and 40.4%, respectively.^[1]^ In a meta-analysis conducted in China, the prevalence of primary osteoporosis was approximately 18.2%, among which the prevalence was 23.4% in women and 11.5% in men.^[3]^ Projections indicate that by 2035, the number of osteoporosis-related fractures in China will reach 4.83 million annually, incurring roughly $19.92 billion in healthcare costs each year, thus representing a significant economic burden.^[4]^

Osteoporotic fractures predominantly affect the forearm, hip, and lumbar spine, with high morbidity and mortality. Approximately 20% to 30% of individuals with osteoporosis succumb within 1 year following an osteoporotic fracture.^[5]^ Given that osteoporosis is typically asymptomatic prior to fracture, early screening and detection are crucial strategies in the management and treatment of this condition.

Osteoporosis was a multifactorial disease, with numerous studies identifying clinical risk factors including age, gender, weight, history of fractures, smoking, alcohol abuse, and corticosteroid use.^[1,6,7]^ Understanding these risk factors is critical for implementing early interventions aimed at preventing the onset and progression of the disease. Previous studies have found that osteoporosis and osteoporotic fractures have significant differences in the incidence rate of men and women. Therefore, we speculate that the relevant non bone metabolism laboratory indicators of osteoporosis may also be different in different genders. Non-bone metabolism laboratory indicators, such as alanine aminotransferase (ALT), aspartate aminotransferase (AST), blood urea nitrogen (BUN), etc, are routine health examination items that are easy to obtain and inexpensive. Confirming this hypothesis could greatly enhance our ability to conduct individual osteoporosis risk assessments and facilitate early prevention and targeted treatment strategies.

## 2. Materials and methods

This study was in accordance with Declaration of Helsinki. The study protocol was approved by the Research Ethics Committee of the Second Hospital of Shandong University (approval number: KYLL2024982). All participants signed a written informed consent form, ensuring their understanding and agreement to participate. Furthermore, the study strictly adhered to principles of confidentiality and anonymity throughout the research process.

### 2.1. Study design

To evaluate the gender differences in osteoporosis risk factors, we collected physical examination data from individuals who underwent physical examinations at the author’s institution’s Health Management Department from January 2022 to June 2024. The inclusion criteria were as follows: age ≥ 50 years; individuals who underwent health examinations, BMD assessments, and related laboratory tests at our institution. The exclusion criteria were: patients who had received anti-osteoporosis treatment for known osteoporosis or osteopenia; a history of metabolic bone disease or chronic conditions affecting calcium absorption, malignancy, use of medications known to influence bone metabolism, and/or confirmed pregnancy; a history of fractures or surgical intervention for fractures; a history of lumbar spine surgery. Based on these inclusion and exclusion criteria, a total of 4343 subjects were included in the study, comprising 2413 males and 1930 females.

### 2.2. Data collection

Height and weight were measured with participants barefoot. Before measuring blood pressure, participants were required to rest quietly for a minimum of 5 minutes, empty their bladder, and abstain from taking any medication that could influence the results. Blood pressure data utilized was the average of 2 readings. Hypertension (per ICD-11 code BA00) was defined as a systolic blood pressure of ≥140 mm Hg and/or a diastolic blood pressure of ≥90 mm Hg, or the use of antihypertensive medication. Type 2 diabetes mellitus was diagnosed according to the WHO criteria (1999),^[8]^ corresponding to ICD-10 code E11.

In order to collect laboratory data, nurses collected blood from the anterior cubital vein in the early morning after the examinee fasted overnight (for at least 8 hours). Professional technicians in the laboratory use Canon TBA-FX8 fully automatic biochemical analyzer to measure ALT, AST, albumin (ALB), alkaline phosphatase (ALP), glutamine transaminase (GGT), creatinine (Cr), uric acid (UA), BUN, total cholesterol (TC), triglycerides (TG), high-density lipoprotein cholesterol (HDL-C), and low-density lipoprotein cholesterol (LDL-C). The hemoglobin concentration (Hb) was measured using XN-9000. The laboratory quality control was carried out in accordance with relevant standards (ISO/IEC 17025:2017).

### 2.3. Assessment of osteoporosis and osteopenia

BMD is the most reliable indicator of fracture risk, with dual-energy X-ray absorptiometry (DXA) being the current gold standard for BMD measurement.^[9]^ Use DXA (Hologic) to measure the BMD of the lumbar spine (L1–L4) and bilateral hip joints of each examinee, and have them assessed daily by the same instrument and professional physician. The BMD values obtained through DXA are expressed as a T-score, which indicate the difference (in standard deviations) between the bone density value of the subject being examined and the average value of a healthy reference population. According to the World Health Organization (WHO) diagnostic criteria for osteoporosis, a T-score of less than −2.5 indicates osteoporosis, a T-score between −1.0 and −2.5 suggests osteopenia, and a T-score of −1.0 or above is considered normal.^[9,10]^

### 2.4. Statistical analysis

Statistical analyses were performed using SPSS 29.0 software. For continuous variables that adhered to a normal distribution, results were presented as the mean ± standard deviation (M ± SD). For those not following a normal distribution, data were reported as mean (25%, 75%). Categorical variables were described using frequencies and percentages. Continuous variables were treated with *t*-test or non-parametric test, while categorical variables were analyzed using chi-square test. Unconditional logistic regression was employed to identify risk factors for osteoporosis and osteopenia, with results expressed as odds ratios (OR) and 95% confidence intervals (CI). A 2-sided *P*-value <.05 was considered statistically significant.

## 3. Results

### 3.1. The characteristics of the study population

**Figure 1.**
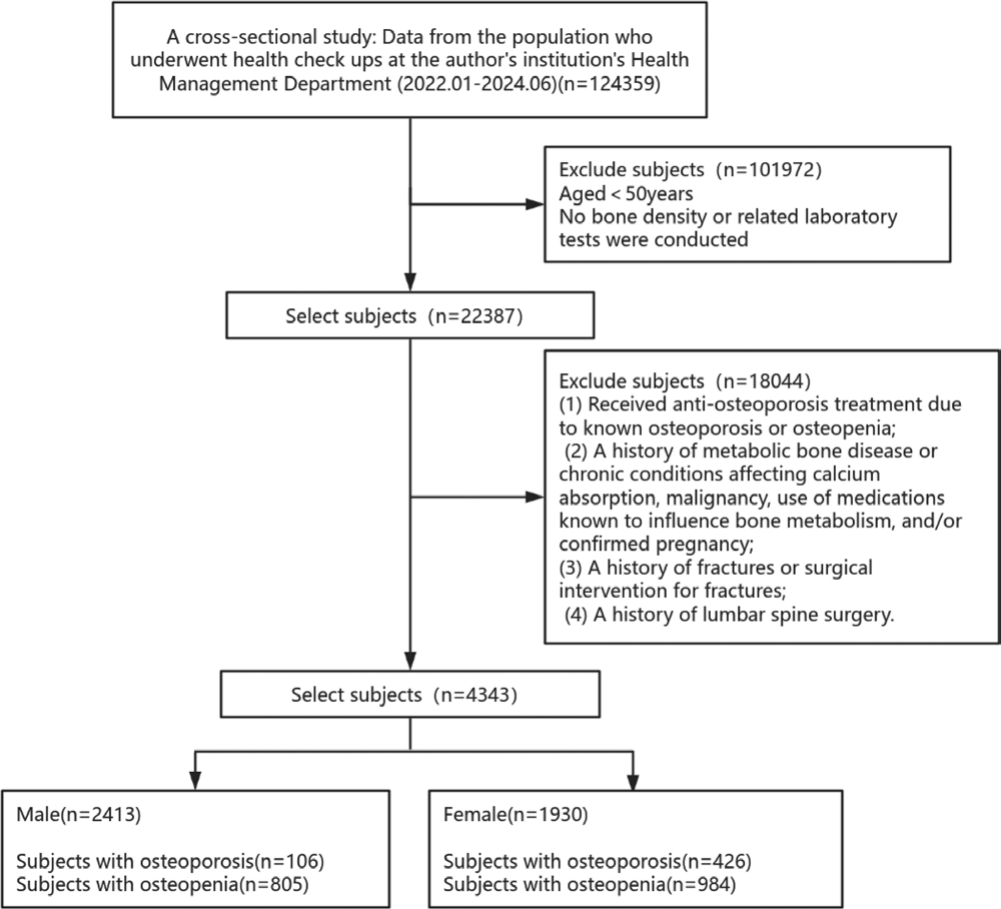
Sample selection procedure used in this study.

Between January 2022 and June 2024, a total of 124,359 patients were evaluated at the institution. As shown in Figure 1, 4343 patients (mean age: 60.15 ± 7.96 years) met the inclusion criteria. The cohort comprised 2413 males and 1930 females, with mean ages of 59.96 ± 8.15 years for males and 60.39 ± 7.72 years for females, respectively. As detailed in Table 1, the overall prevalence of osteoporosis was found to be 12.2%, with the condition affecting 4.4% of males and 22.1% of females. Additionally, osteopenia was identified in 41.2% of the patients, including 33.36% of males and 50.98% of females. The incidence of both osteoporosis and osteopenia was significantly higher in females compared to males (*P* < .05). Conversely, the prevalence of hypertension and diabetes was significantly higher in males than in females (*P* < .05).

**Table 1:** General characteristics of the population undergoing health check ups.

	Male (2413)	Female (1930)	*P*-value
Age (yr)	59.96 ± 8.15	60.39 ± 7.72	.003*
Height (cm)	170.34 ± 5.83	158.80 ± 5.32	<.001*
Weight (kg)	75.29 ± 10.62	62.68 ± 9.09	<.001*
Hypertension (%)	1525 (63.20%)	926 (49.84%)	<.001*
Diabetes (%)	485 (20.10%)	262 (13.58%)	<0.001*
ALT (U/L)	22.65 ± 12.80	19.21 ± 9.55	–
AST (U/L)	20.66 ± 7.58	19.93 ± 5.92	–
ALB (g/L)	46.28 ± 2.43	45.76 ± 2.28	<.001*
ALP (U/L)	70.71 ± 18.20	77.71 ± 19.44	<.001*
GGT(U/L)	33.22 ± 32.71	20.65 ± 12.41	<.001*
Cr (μmol/L)	73.01 ± 11.42	56.47 ± 8.36	<.001*
UA (μmol/L)	350.68 ± 5.16	284.41 ± 64.35	<.001*
BUN (mmol/L)	5.16 ± 4.98	4.85 ± 1.14	<.001*
TC (mmol/L)	4.98 ± 0.99	5.40 ± 0.98	<.001*
TG (mmol/L)	1.54 ± 1.26	1.41 ± 0.87	.059
HLD-C (mmol/L)	1.23 ± 0.26	1.39 ± 0.26	<.001*
LDL-C (mmol/L)	2.84 ± 0.85	3.08 ± 0.86	<.001*
HB (g/L)	151.95 ± 10.88	133.62 ± 9.88	–
Osteoporosis			
Non-OP (%)	2307 (95.6%)	1504 (77.9%)	<.001*
OP (%)	106 (4.4%)	426 (22.1%)	<.001*
Osteopenia			
Normal (%)	1608 (66.64%)	946 (49.02%)	<.001*
OPA (%)	805 (33.36%)	984 (50.98%)	<.001*

Continuous variables were represented as the mean ± standard deviation. Categorical variables were described using frequencies and percentages.

ALB = albumin, ALP = alkaline phosphatase, ALT = alanine aminotransferase, AST = aspartate aminotransferase, BUN = blood urea nitrogen, Cr = creatinine, GGT = glutamine transaminase, HB = hemoglobin concentration, HLD-C = high-density lipoprotein cholesterol, LDL-C = low-density lipoprotein cholesterol, Non-OP = non-osteoporosis, OP = osteoporosis, OPA = osteopenia, TC = total cholesterol, TG = triglycerides, UA = uric acid.

* *P* < .05.

### 3.2. Non-bone metabolic indicators

As shown in Table 2, there are significant differences in age, height, weight, ALT, ALB, ALP, AST, Cr, UA, BUN, TG, and Hb between male osteoporosis and non- osteoporosis populations (*P* < .05). In men with normal bone density and those with osteopenia, significant differences were noted in age, height, weight, ALT, ALB, ALP, Cr, UA, BUN, TG, HDL-C, and Hb (*P* < .05). Nonetheless, there was no significant difference in the prevalence of hypertension and diabetes between men with osteoporosis and non-osteoporosis, as well as those with normal BMD and osteopenia.

**Table 2:** Male non-bone metabolic laboratory indicators.

Characteristics	No-osteoporosis	Osteoporosis	*P*-value	No-osteopenia	Osteopenia	*P*-value
Age (yr)	59.58 ± 7.83	68.08 ± 10.40	<.001	58.86 ± 7.41	62.13 ± 9.06	<.001*
Height (cm)	170.48 ± 5.79	167.17 ± 5.86	<.001	170.76 ± 5.63	169.49 ± 6.13	<.001*
Weight (kg)	75.63 ± 10.47	67.68 ± 11.02	<.001	76.64 ± 10.57	72.57 ± 10.19	<.001*
Hypertension (%)	1454 (63.03%)	71 (66.98%)	.409	1007 (62.62%)	518 (64.35%)	.408
Diabetes (%)	461 (19.98%)	24 (22.64%)	.504	312 (19.40%)	173 (21.39%)	.228
ALT (U/L)	22.79 ± 12.68	19.63 ± 14.82	<.001	23.09 ± 13.08	21.78 ± 12.17	.002*
AST (U/L)	20.63 ± 7.23	21.36 ± 13.15	.727	20.60 ± 7.21	20.78 ± 8.28	.611
ALB (g/L)	46.31 ± 2.41	45.68 ± 2.79	.013	46.36 ± 2.37	46.13 ± 2.54	.045*
ALP (U/L)	70.52 ± 18.20	74.87 ± 17.61	.005	69.27 ± 17.73	73.60 ± 18.77	<.001*
GGT (U/L)	33.37 ± 32.88	29.77 ± 28.71	.037	33.24 ± 33.07	33.17 ± 32.01	.884
Cr (μmol/L)	73.14 ± 11.06	70.30 ± 17.47	<.001	73.48 ± 11.02	72.07 ± 12.15	<.001*
UA (μmol/L)	351.74 ± 76.71	327.52 ± 72.89	.003	353.80 ± 76.70	344.44 ± 76.34	.012*
BUN (mmol/L)	5.16 ± 1.17	4.99 ± 1.40	.023	5.20 ± 1.18	5.06 ± 1.18	.009*
TC (mmol/L)	4.98 ± 0.99	4.93 ± 1.13	.676	4.99 ± 0.96	4.96 ± 1.05	.389
TG (mmol/L)	1.55 ± 1.27	1.25 ± 0.86	.001	1.59 ± 1.30	1.44 ± 1.15	.004*
HLD-C (mmol/L)	1.22 ± 0.25	1.29 ± 0.39	.277	1.22 ± 0.24	1.25 ± 0.28	.034*
LDL-C (mmol/L)	2.85 ± 0.84	2.82 ± 0.92	.815	2.84 ± 0.83	2.86 ± 0.89	.670
HB (g/L)	152.15 ± 10.71	147.55 ± 13.37	.01	152.59 ± 10.62	150.67 ± 11.28	<.001*

Continuous variables were represented as the mean ± standard deviation. Categorical variables were described using frequencies and percentages.

ALB = albumin, ALP = alkaline phosphatase, ALT = alanine aminotransferase, AST = aspartate aminotransferase, BUN = blood urea nitrogen, Cr = creatinine, GGT = glutamine transaminase, HB = hemoglobin concentration, HLD-C = high-density lipoprotein cholesterol, LDL-C = low-density lipoprotein cholesterol, TC = total cholesterol, TG = triglycerides, UA = uric acid.

* *P* < .05.

Table 3 demonstrates statistically significant differences among women in various parameters, including age, height, weight, hypertension, diabetes, ALT, ALB, ALP, and UA (*P <* .05). Furthermore, among women categorized by normal BMD and those with osteopenia, significant differences were observed in age, height, weight, hypertension, diabetes, ALT, ALB, ALP, UA, BUN, TC, and HDL-C, with all differences also achieving statistical significance (*P* < .05).

**Table 3:** Female non-bone metabolic laboratory indicators.

Characteristics	No-osteoporosis	Osteoporosis	*P*-value	No-osteopenia	Osteopenia	*P*-value
Age (yr)	58.97 ± 7.01	65.39 ± 8.02	<.001*	57.52 ± 6.47	63.15 ± 7.82	<.001*
Height (cm)	159.28 ± 5.20	157.08 ± 5.41	<.001*	159.65 ± 5.25	157.97 ± 5.27	<.001*
Weight (kg)	63.46 ± 9.10	59.93 ± 8.51	<.001*	64.46 ± 9.22	60.96 ± 8.62	<.001*
Hypertension (%)	714 (47.47%)	248 (58.22%)	<.001*	424 (44.82%)	538 (54.67%)	<.001*
Diabetes (%)	189 (12.57%)	73 (17.14%)	0.015*	107 (11.31%)	155 (15.75%)	.004*
ALT (U/L)	19.63 ± 9.80	17.75 ± 8.46	<.001*	19.83 ± 9.99	18.61 ± 9.06	.002*
AST (U/L)	20.02 ± 5.91	19.60 ± 5.93	.109	20.01 ± 5.97	19.85 ± 5.86	.342
ALB (g/L)	45.86 ± 2.27	45.41 ± 2.28	.001*	45.89 ± 2.27	45.64 ± 2.28	.032*
ALP (U/L)	76.15 ± 18.73	83.21 ± 20.86	<.001*	74.72 ± 18.22	80.58 ± 20.14	<.001*
GGT (U/L)	20.85 ± 12.54	19.96 ± 11.95	.083	20.76 ± 12.94	20.54 ± 11.89	.881
Cr (μmol/L)	56.46 ± 8.24	56.48 ± 8.80	.996	56.19 ± 8.15	56.73 ± 8.56	.210
UA (μmol/L)	288.00 ± 64.76	271.74 ± 61.31	<.001*	288.82 ± 66.27	280.17 ± 62.19	.003*
BUN (mmol/L)	4.83 ± 1.12	4.91 ± 1.20	.297	4.74 ± 1.09	4.95 ± 1.17	<.001*
TC (mmol/L)	5.39 ± 0.97	5.43 ± 1.03	.481	5.33 ± 0.96	5.46 ± 1.00	.002*
TG (mmol/L)	1.42 ± 0.88	1.37 ± 0.83	.199	1.40 ± 0.85	1.42 ± 0.89	.829
HLD-C (mmol/L)	1.38 ± 0.26	1.40 ± 0.26	.168	1.37 ± 0.25	1.41 ± 0.26	.002*
LDL-C (mmol/L)	3.09 ± 0.85	3.07 ± 0.91	.569	3.06 ± 0.84	3.11 ± 0.89	.226
HB (g/L)	133.77 ± 9.74	133.10 ± 10.34	.426	133.86 ± 10.11	133.40 ± 9.64	.215

Continuous variables were represented as the mean ± standard deviation. Categorical variables were described using frequencies and percentages.

ALB = albumin, ALP = alkaline phosphatase, ALT = alanine aminotransferase, AST = aspartate aminotransferase, BUN = blood urea nitrogen, Cr = creatinine, GGT = glutamine transaminase, HB = hemoglobin concentration, HLD-C = high-density lipoprotein cholesterol, LDL-C = low-density lipoprotein cholesterol, TC = total cholesterol, TG = triglycerides, UA = uric acid.

* *P* < .05.

### 3.3. Logistic regression model for non-bone metabolic indicators

Tables 4 and 5 indicate an increasing prevalence of osteoporosis and osteopenia with advancing age. In men aged 60 to 69, the risk of developing osteoporosis and osteopenia was 3.46 and 1.81 times higher, respectively, compared to those aged 50 to 59. For men above the age of 70, the risk increased to 9.48 times for osteoporosis and 2.79 times for osteopenia, in comparison to the 50 to 59 age group. In women, the risk of osteoporosis at 60 to 69 years of age was 3.34 times higher, while the risk in those over 70 was 7.11 times higher, when compared to women aged 50 to 59.

**Table 4:** Logistic regression model of non-bone metabolic laboratory indicators related to male and female osteoporosis.

Characteristics	Osteoporosis (male)			Osteoporosis (female)		
	OR	95% CI	*P*-value	OR	95% CI	*P*-value
Age (yr)						
50–59	Reference			Reference		
60–69	3.458	2.000–5.978	<.001*	3.343	2.567–4.354	<.001*
70+	9.478	5.135–17.493	<.001*	7.105	4.950–10.199	<.001*
Height (cm)	0.989	0.950–1.031	.612	0.983	0.958–1.007	.166
Weight (kg)	0.942	0.918–0.967	<.001*	0.963	0.948–0.978	<.001*
Hypertension (%)	–	–	–	1.228	0.945–1.595	.125
Diabetes (%)	–	–	–	1.006	0.723–1.400	.971
ALT (U/L)	0.994	0.973–1.015	.575	0.986	0.972–1.000	.046*
ALB (U/L)	1.020	0.934–1.113	.662	0.968	0.917–1.021	.225
ALP (U/L)	1.012	1.002–1.023	.025*	1.021	1.014–1.027	<.001*
GGT (U/L)	1.002	0.995–1.008	.610			
UA (μmol/L)	1.001	0.998–1.004	.677	0.996	0.994–0.998	<.001*
BUN (mmol/L)	0.822	0.682–0.992	.041*	–	–	–
TG (mmol/L)	0.906	0.712–1.154	.425	–	–	–
HB (g/L)	0.994	0.976–1.013	.559	–	–	–
Cr (μmol/L)	0.983	0.963–1.004	0.104			

A 2-sided *P*-value < .05 was considered statistically significant. There is a linear relationship between the logit transformation values of continuous variables and the dependent variable, so not all continuous variables have been transformed into ordered categorical variables, except for age.

95% CI = 95% confidence intervals, ALB = albumin, ALP = alkaline phosphatase, ALT = alanine aminotransferase, BUN = blood urea nitrogen, GGT = glutamine transaminase, HB = hemoglobin concentration, OR = odds ratios, TG = triglycerides, UA = uric acid.

* *P* < .05.

**Table 5:** Logistic regression model of non-bone metabolic laboratory indicators related to male and female osteopenia.

Characteristics	Osteopenia (male)			Osteopenia (female)		
	OR	95% CI	*P*-value	OR	95% CI	*P*-value
Age (yr)						
50–59	Reference		<.001*	Reference		
60–69	1.808	1.483–2.204	<.001*	3.344	2.701–4.141	<.001*
70+	2.792	2.070–3.767	<.001*	5.370	3.721–7.750	<.001*
Height (cm)	1.009	0.990–1.027	.363	0.995	0.975–1.016	.662
Weight (kg)	0.965	0.955–0.976	<.001*	0.957	0.945–0.970	<.001*
Hypertension (%)	–	–	–	1.306	1.051–1.623	.016*
Diabetes (%)	–	–	–	1.076	0.797–1.453	.631
ALT (U/L)	0.999	0.991–1.006	.738	0.996	0.985–1.007	.442
ALB (U/L)	1.009	0.971–1.049	.632	0.970	0.927–1.014	.179
ALP (U/L)	1.014	1.009–1.019	<.001*	1.018	1.013–1.024	<.001*
UA (μmol/L)	1.001	1.000–1.002	.106	0.998	0.997–1.000	.030*
BUN (mmol/L)	0.876	0.808–0.950	.001*	1.150	1.052–1.258	.002*
TC (mmol/L)				1.156	1.031–1.296	.013*
HLD-C (mmol/L)	1.080	0.749–1.559	.679	1.238	0.798–1.922	.341
TG (mmol/L)	0.959	0.884–1.039	.306	–	–	–
HB (g/L)	0.995	0.986–1.004	.276	–	–	–
Cr (μmol/L)	0.992	0.983–1.001	.070	–	–	–

A 2-sided *P*-value <.05 was considered statistically significant. There is a linear relationship between the logit transformation values of continuous variables and the dependent variable, so not all continuous variables have been transformed into ordered categorical variables, except for age.

95% CI = 95% confidence intervals, ALB = albumin, ALP = alkaline phosphatase, ALT = alanine aminotransferase, BUN = blood urea nitrogen, Cr = creatinine, GGT = glutamine transaminase, HB = hemoglobin concentration, HLD-C = high-density lipoprotein cholesterol, OR = odds ratios, TG = triglycerides, UA = uric acid.

* *P* < .05.

As indicated in Table 4, among men, weight (OR, 0.942; 95% CI, 0.918–0.967; *P* < .001), ALP (OR, 1.012; 95% CI, 1.002–1.023; *P* = .025), and BUN (OR, 0.822; 95% CI, 0.682–0.992; *P* = .041) are significantly associated with osteoporosis. In women, weight (OR, 0.963; 95% CI, 0.948–0.978; *P* < .001), ALT (OR, 0.986; 95% CI, 0.972–1.000; *P* = .046), ALP (OR, 1.021; 95% CI, 1.014–1.027; *P* < .001), and UA (OR, 0.996; 95% CI, 0.994–0.998; *P* < .001) are correlated with osteoporosis.

In Table 5, for men, weight (OR, 0.965; 95% CI, 0.955–0.976; *P* < .001), ALP (OR, 1.014; 95% CI, 1.009–1.019; *P* < .001), and BUN (OR, 0.876; 95% CI, 0.808–0.950; *P* = .001) demonstrate a correlation with osteopenia. Among women, weight (OR, 0.957; 95% CI, 0.945–0.970; *P* < .001), hypertension (OR, 1.306; 95% CI, 1.051–1.623; *P* = .016), ALP (OR, 1.018; 95% CI, 1.013–1.024; *P* < .001), UA (OR, 0.998; 95% CI, 0.997–1.000; *P* = .030), BUN (OR, 1.150; 95% CI, 1.052–1.258; *P* = .002), and TC (OR, 1.156; 95% CI, 1.031–1.296; *P* = .013) are associated with osteopenia.

**Figure 2.**
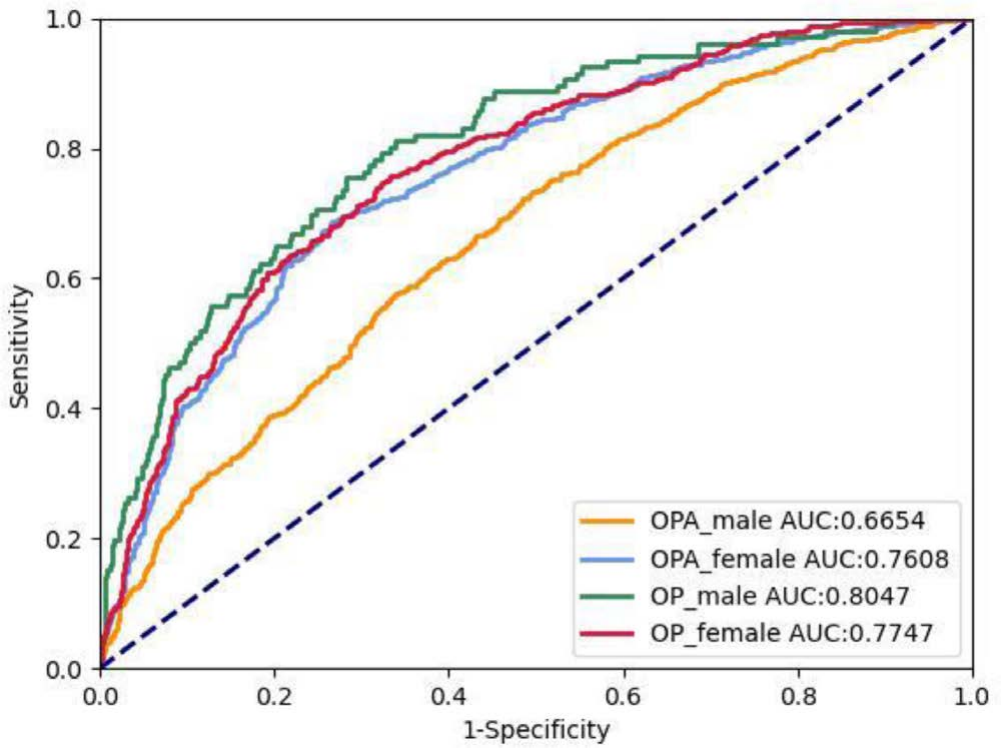
Logistic receiver operating characteristic (ROC) curves for osteoporosis and osteoporosis. OP = osteoporosis, OPA = osteopenia.

Figure 2 shows the logistic receiver operating characteristic (ROC) curves for osteoporosis and osteopenia. The AUC of the male osteoporosis prediction model is 0.805, while the AUC of the female osteoporosis prediction model is 0.775. The AUC of the male Osteopenia prediction model is 0.665, and the AUC of the female Osteopenia prediction model is 0.761.

### 3.4. Variable importance

**Figure 3.**
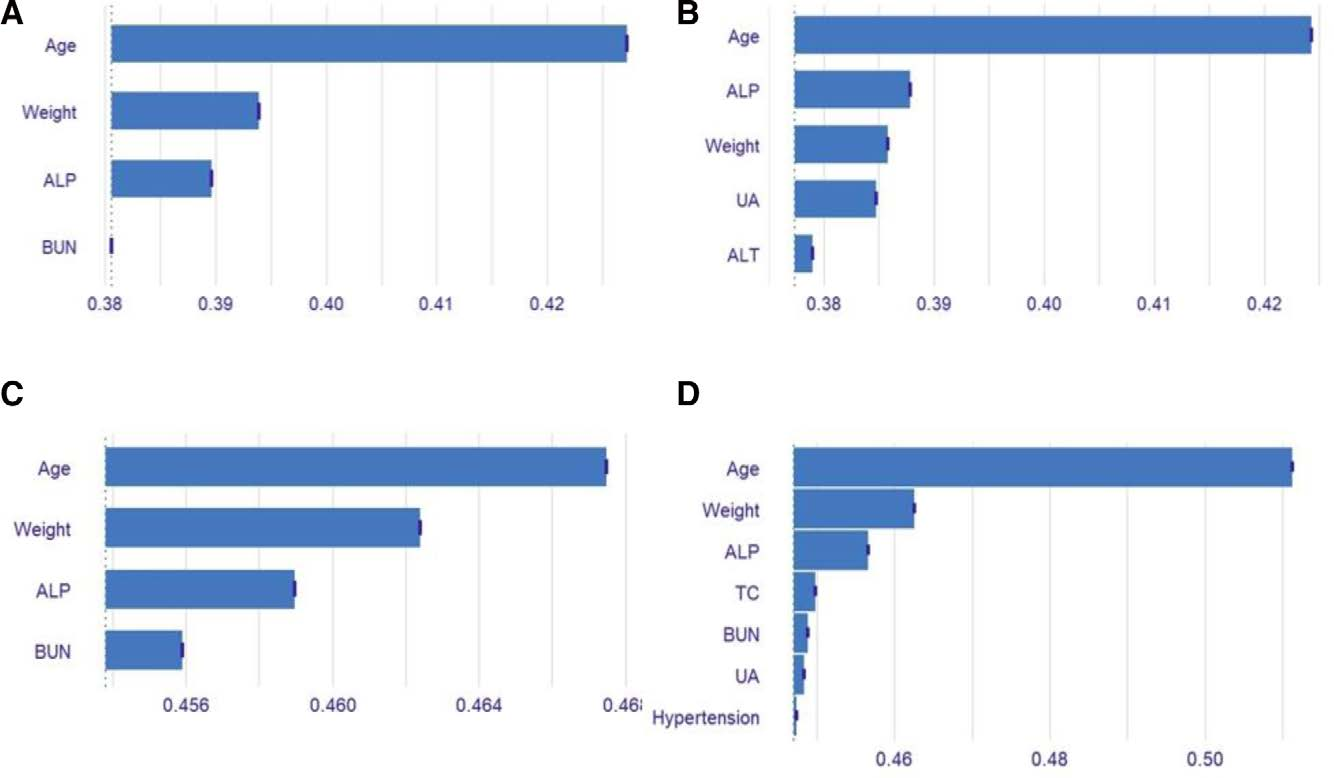
Variable importance derived from the logistic regression. (A) Variable importance of male osteoporosis. (B) Variable importance of female osteoporosis. (C) Variable importance of male Osteopenia. (D) Variable importance of female Osteopenia. ALP = alkaline phosphatase, ALT = alanine aminotransferase, BUN = blood urea nitrogen, TC = total cholesterol, UA = uric acid.

Use the DALEX package in RStudio to analyze how different feature variables affect osteoporosis and Osteopenia using logistic regression. The variable importance is illustrated in Figure 3. Among the factors affecting male osteoporosis, the variables are ranked by importance as follows: age, weight, ALP, and BUN. For female osteoporosis, the variables are ranked by importance as follows: age, ALP, weight, and ALT. The following variables are ranked by their importance in male Osteopenia: age, weight, ALP, and BUN. For female Osteopenia, the variables are ranked by importance as follows: age, weight, ALP, TC, BUN, UA, and hypertension.

## 4. Discussion

The findings of this study suggest that the common influencing factors of osteoporosis are age, weight, ALP. In men, BUN is identified as an influencing factor, whereas, in women, ALT and UA are influencing factors. The influencing factors of female osteopenia are age, weight, ALP, and BUN. For women, influencing factors of osteopenia are hypertension, UA, and TC. Notably, the factors influencing both osteoporosis and osteopenia are consistent in the male population. However, distinct differences are observed in the factors influencing osteoporosis and osteopenia within the female population.

Age and ALP are established risk factors for osteoporosis and osteopenia in both male and female populations, whereas weight serves as a protective factor. The prevalence of osteoporosis and osteopenia escalates with advancing age, with women experiencing a significantly higher incidence compared to men, aligning with previously published studies.^[4,11,12]^ The aging process is associated with a decline in physiological functions, resulting in diminished absorption of calcium and vitamin D, as well as a decrease in bone mass formation.^[13]^ ALP is an enzyme present in several organs, including the liver, bile ducts, kidneys, and bones. It is primarily associated with osteoblast activity in bone metabolism, playing a significant role in osteoid formation and bone mineralization.^[14]^ When serum ALP levels rise, it suggests heightened osteoblastic activity, reduced synthesis of bone cells, and a compromised ability to effectively support the production of new bone matrix, ultimately resulting in diminished bone strength.^[15]^ A cross-sectional study involving 6331 young adults demonstrated an inverse relationship between serum total ALP levels and BMD.^[16]^ Weinreb et al developed a cholestatic liver disease model in rats through bile duct ligation (BDL) and observed elevated ALP levels. Their findings indicated that BDL rats exhibited fragile bones and diminished osteoblast numbers, which contributed to decreased maximum bone tensile strength and stiffness.^[17]^ Naylor and Eastell’s research further corroborated the association between increased ALP activity and elevated bone turnover.^[18]^ Chuang et al have corroborated that age and ALP are significant negative determinants of BMD, a pattern observed consistently across various measurement sites in both male and female populations, aligning with our findings.^[19]^ Greco et al reported that overweight status may have either a neutral or protective role concerning BMD.^[20]^ In a longitudinal study by Chiu et al, involving 24,507 participants over an average duration of 47 months, it was demonstrated that individuals classified as underweight exhibited a higher risk of osteoporosis compared to those with normal weight.^[21]^ Underweight was identified as an independent risk factor for osteoporosis. This finding is also in agreement with our research, potentially attributable to the biomechanical load imposed by body weight on bones. Increased body weight applies greater static mechanical stress on the skeletal system, enhancing bone density and consequently reducing the incidence of osteoporosis among the elderly.^[22]^ Nonetheless, the precise underlying biological mechanisms remain to be fully elucidated.

This research has found that the influencing factors of male osteoporosis are BUN, while of females, are ALT and UA. BUN was identified as a protective factor against osteoporosis and osteopenia in males, whereas it was associated with an increased risk of osteopenia in females. The kidneys play a pivotal role in maintaining calcium and phosphorus homeostasis. Serum creatinine and BUN are well-established indicators for assessing renal function. Renal function decreases with age. Approximately 20% of American adults aged 65 and older experience moderate to severe renal dysfunction.^[23]^ A glomerular filtration rate below 60 mL/min/1.73 m^2^ results in phosphate retention, which subsequently elevates parathyroid hormone levels and reduces 1,25-dihydroxyvitamin D concentrations, both of which adversely affect BMD.^[24]^ Subclinical renal insufficiency is a prevalent condition that often eludes detection through standard clinical chemistry assessments, such as BUN or Cr measurements. According to the study by Hsu et al, while individuals with renal insufficiency exhibited significantly reduced BMD, the analysis, after adjusting for confounding variables, revealed that renal function alone was not independently associated with alterations in BMD. Furthermore, mild to moderate chronic renal insufficiency did not result in a decrease in BMD.^[25]^ According to the study by Hsu et al, while individuals with renal insufficiency exhibited significantly reduced BMD, the analysis, after adjusting for confounding variables, revealed that renal function alone was not independently associated with alterations in BMD. Furthermore, mild to moderate chronic renal insufficiency did not result in a decrease in bone density.^[25]^ Park et al evaluated the relationship between renal function and BMD in healthy Korean women. In logistic regression analysis, BUN and cystatin C were associated with lumbar and femoral BMD. After adjusting for age, menopause, and body mass index (BMI), only creatinine was negatively correlated with lumbar BMD.^[26]^ At present, the mechanism by which BUN influences BMD remains unclear, presenting an opportunity for future research.

Our study further corroborates the role of UA as a protective factor against osteoporosis and osteopenia in women, a finding that does not extend to men. Yan et al demonstrated that UA exerts a protective effect specifically in postmenopausal women. In contrast, in men, UA does not correlate with osteoporosis risk, though it is significantly associated with BMD at the femoral neck and total hip joints.^[27]^ These results align closely with the findings of our research. Li et al used multivariate linear regression analyses to evaluate the relationship between serum uric acid (SUA) and lumbar spine BMD in American men. After completely adjusting for potential confounding factors, they found no correlation between male SUA and lumbar spine BMD.^[28]^ Xu et al found that SUA levels are independently positively correlated with BMD in patients with osteoporosis. However, in normal and low body weight osteoporosis patients, SUA levels below 296 μmol/L may have a protective effect on BMD, while SUA levels above this concentration are not associated with BMD.^[29]^ Some studies attribute the protective effect of UA on osteoporosis to its antioxidant activity. Previous research has established a link between reduced BMD and environments characterized by oxidative stress and inflammation.^[30]^ UA is an endogenous antioxidant that can easily eliminate free radicals in plasma, especially under oxidative stress conditions, and has a protective effect on osteoporosis.^[31,32]^ Although correcting hyperuricemia is important for reducing the risk of other types of diseases, attention should be paid to preventing excessive correction and avoiding mitigating the beneficial effects of SUA as an oxidative stress inhibitor. Therefore, after a comprehensive assessment of the risk of osteoporosis and other diseases in patients, it is necessary to determine the optimal level of SUA control. ALT is a crucial biomarker for liver function, with elevated serum ALT levels indicative of hepatic impairment.^[33]^ It is worth noting that research conducted in the past 10 years has shown that the skeletal joint system is not just a simple load-bearing structure, but also an important endocrine organ that secretes cytokines that govern many organs throughout the body, including the liver.^[34]^ A prospective cohort study involving 2055 community participants revealed that women with lower serum osteocalcin levels are at an increased risk of developing nonalcoholic fatty liver disease. Further animal studies have demonstrated that short-term osteocalcin treatment in mice can mitigate hepatic steatosis.^[35]^ Furthermore, bone harbors a substantial population of mesenchymal stem cells, which have been shown to modulate CD4 T cell differentiation in murine models, consequently alleviating nonalcoholic liver steatosis.^[36]^ Although there is currently no clear research on the relationship between BMD and liver enzymes, the above indirect findings may help partially explain the relationship between BMD and ALT.

This study identified a correlation between hypertension and an elevated risk of Osteopenia in women, although this association was not observed in men suffering from osteoporosis and osteopenia. Previous research has substantiated the existence of potential shared risk factors between hypertension and osteoporosis, including metabolic factors such as secondary hyperparathyroidism, increased sympathetic nervous system activity, oxidative stress, inhibition of vitamin K-dependent matrix proteins, osteopontin, and angiotensin II, as well as associated molecular mechanisms like histone modifications.^[37–39]^ Additionally, some studies have demonstrated that individuals with cardiovascular disease are at a heightened risk of experiencing bone loss.^[40]^ Osteoporosis and hypertension are prevalent age-associated pathologies. In women, fluctuations in estrogen levels due to menopause or aging can significantly impact both vascular integrity and bone metabolism.^[41]^ Estrogen is recognized as a crucial regulator of both osseous and vascular systems, enhancing endothelial cell and vascular smooth muscle cell functionality, inhibiting platelet aggregation, and modulating vascular injury responses.^[42]^ Furthermore, circulating estrogen plays a critical role in osteoclast regulation by reducing their number and activity, thereby inhibiting bone resorption and attenuating bone remodeling processes while preserving bone formation. Consequently, diminished estrogen levels result in augmented bone resorption.^[43,44]^ This mechanistic pathway elucidates the observed association in our study whereby hypertension correlates with an elevated risk of bone loss in women, a pattern not observed in their male counterparts.

Research has found that the influencing factors of male osteopenia are BUN, and in females, are hypertension, UA, and TC. The roles of BUN and UA have been discussed previously. Lian et al reported that TC and LDL-C are risk factors for osteoporosis, whereas HDL-C and weight serve as protective factors against osteoporosis.^[45]^ In a study conducted by Hsu et al involving 7137 Chinese men and 4585 premenopausal and 2248 postmenopausal women, a significant negative correlation was observed between systemic bone mineral content (BMC) and levels of cholesterol, TG, LDL, and the LDL/HDL ratio. However, no significant correlation was found with HDL levels.^[46]^ Another study showed that elderly women with high BMI and high fat content have an increased conversion of body fat to estrogen synthesis, which can inhibit the action of osteoclasts. The increased fat cells stimulate insulin secretion, which has a positive effect on the formation of osteoblasts and promotes an increase in BMD. Our research results indicate that TC is a risk factor for osteopenia in women, but it has no effect on osteoporosis. The possible mechanism is that estrogen has a protective effect on BMD, but lipid metabolism disorders can affect estrogen synthesis and thus affect the occurrence and development of osteoporosis. Dysregulation of lipid metabolism in postmenopausal women is closely related to bone health dysfunction.^[47]^ The proposed mechanism involves adipose tissue serving as a primary source of estrogen, synthesized within these cells via the aromatization of androgens. Estrogen plays a crucial role in regulating osteoclast apoptosis, and disturbances in lipid metabolism can influence estrogen synthesis, consequently impacting the onset and progression of osteoporosis. Estrogen therapy for postmenopausal osteoporosis has demonstrated efficacy in reducing levels of TC and LDL-C.^[48,49]^

The analysis indicates that the impact of the same influencing factor on osteoporosis and osteopenia varies between genders. Even within the same gender, the effects on osteoporosis and osteopenia differ. Age is the most significant factor in osteoporosis and osteopenia across different genders, consistent with previous studies that highlight age as a crucial risk factor for osteoporosis.^[50,51]^ The analysis shows that, in males, weight influences osteoporosis more than ALP, while the reverse is true in females.^[52,53]^ This finding suggests that, aside from the uncontrollable factor of age, prevention strategies for osteoporosis should differ between males and females. The analysis reveals that, in men, weight and age contribute equally to osteopenia, whereas, in women, age plays a much more significant role. For men, maintaining a healthy weight is crucial in preventing osteopenia. For women, the reason why age has a greater impact may be related to estrogen levels. After menopause, ovarian function decreases, leading to lower estrogen levels. This decline makes it harder to inhibit osteoclast activity, resulting in rapid bone cell decomposition and absorption, which accelerates bone loss and raises the risk of osteopenia or osteoporosis.^[43]^

In conclusion, there are discernible differences in non-bone metabolic laboratory indicators associated with osteoporosis between males and females. The identification of these differences holds potential for enhancing the prediction of osteoporosis in distinct genders and guiding the development of tailored prevention strategies. Nonetheless, our research is subject to several limitations. Firstly, being a cross-sectional study, it does not establish causal relationships between influencing factors and osteoporosis. Secondly, lifestyle habits such as smoking, drinking, sleep, and diet may have an impact on bone metabolism, which was not taken into account in this study. Thirdly, this is a small-scale single center study, and due to the inherent individual variability across different ethnic groups, the generalizability of our findings to other populations and regions remains uncertain. Finally, our results may be influenced by certain biases, including survey bias and the reliance on self-reported diagnoses of osteoporosis. Therefore, in the future, we will consider conducting further large-scale prospective cohort studies.

## 5. Conclusion

The laboratory indicators associated with non-bone metabolism and their relation to osteoporosis exhibit gender-specific variations. The influencing factors of osteoporosis in males are BUN, and in females, ALT and UA. The influencing factors of osteopenia in males are BUN, while in females, they are hypertension UA, TC. Age, weight, and ALP are common influencing factors for osteoporosis and osteopenia in different genders, but the importance of weight and ALP varies. The importance of weight was almost equal to age in male with osteopenia, but was significantly less than that of age in female. The importance ranking of influencing factors in male osteoporosis is weight and ALP, while for females it is ALP and weight. Discovering these differences can help develop different prevention and treatment strategies for individuals with osteopenia or osteoporosis of different genders.

## Author contributions

**Conceptualization:** Yixin Li.

**Data curation:** Xiaoqian Kong, Bohan Li, Yan Shi.

**Formal analysis:** Xiaoqian Kong.

**Investigation:** Xiaoqian Kong, Bohan Li, Zhe Fang.

**Methodology:** Bohan Li.

**Project administration:** Yixin Li, Yan Shi.

**Software:** Bohan Li.

**Supervision:** Yixin Li.

**Validation:** Yixin Li.

**Visualization:** Yan Shi, Zhe Fang.

**Writing – original draft:** Xiaoqian Kong, Bohan Li.

**Writing – review & editing:** Yixin Li.
